# Nutritional Vitamin D in Renal Transplant Patients: Speculations and Reality

**DOI:** 10.3390/nu9060550

**Published:** 2017-05-27

**Authors:** Piergiorgio Messa, Anna Regalia, Carlo Maria Alfieri

**Affiliations:** 1 Fondazione IRCCS Ca’ Granda Ospedale Maggiore Policlinico, Milano 20122, Italy; carlo.alfieri@policlinico.mi.it; 2 via Festa del Perdono, Università degli Studi di Milano, Milano 20122, Italy; anna.regalia@unimi.it

**Keywords:** vitamin D, calcifediol, VDR, renal transplantation, CKD

## Abstract

Reduced levels of nutritional vitamin D are commonly observed in most chronic kidney disease (CKD) patients and particularly in patients who have received a kidney transplant (KTx). In the complex clinical scenario characterizing the recipients of a renal graft, nutritional vitamin D deficiency has been put in relation not only to the changes of mineral and bone metabolism (MBM) after KTx, but also to most of the medical complications which burden KTx patients. In fact, referring to its alleged pleiotropic (non-MBM related) activities, vitamin D has been claimed to play some role in the occurrence of cardiovascular, metabolic, immunologic, neoplastic and infectious complications commonly observed in KTx recipients. Furthermore, low nutritional vitamin D levels have also been connected with graft dysfunction occurrence and progression. In this review, we will discuss the purported and the demonstrated effects of native vitamin D deficiency/insufficiency in most of the above mentioned fields, dealing separately with the MBM-related and the pleiotropic effects.

## 1. Introductory Notes

Vitamin D has long been recognized as one of the main factors involved in the regulation of calcium and phosphorus metabolism and in the development and maintenance of the structure and function of the musculo-skeletal system [1,2,3].

In humans the body pool of vitamin D is mainly supplied by its endogenous synthesis which occurs in the skin, and by diet contribution [4].

The endogenous pathway begins in the liver where pro-vitamin D (7-dehydrocholesterol) is synthesized from cholesterol; thereafter, 7-dehydrocholesterol is transformed in pre-vitamin D3 (cholecalciferol) in the skin under exposure to UVB light. The endogenous pathway, which is the major source of vitamin D, supplies only vitamin D3, while dietary sources can supply either vitamin D2 (ergocalciferol), mainly contained in plant products, or vitamin D3, contained in animal derived foods (fish, meat, dairy products) [2,4]. The native forms of vitamin D are first converted to 25(OH)D (calcidiol/calcifediol) in the liver which circulates in the blood, mainly bound to a specific binding protein (DBP) and by a far lesser extent to albumin. Then, 25(OH)D is hydroxylated at the 1-α position in the kidney by a specific hydroxylase (CYP27B1) and converted into the most active natural vitamin D metabolite (1,25(OH)2D, calcitriol). Calcitriol is then secreted in the general circulation and reaches its main target organs (intestine, bone, and parathyroid cells), where it exerts its biological classical effects related to the mineral and bone metabolism, through both the genomic (action at specific vitamin D responsive sequences of DNA) and non-genomic (direct effects at cytoplasmic membrane and intra-cytosolic levels) pathways after binding with specific vitamin D receptors (VDR) and possibly other less defined receptors [5]. In recent decades, many experimental studies clearly demonstrated that both the synthesis of active vitamin D and the expression of VDR are not confined to the kidney and its target organs, respectively, but are also present in many other tissues [6,7,8]. Consequently, there was an increasing awareness that vitamin D metabolites have not only an endocrine role mainly devoted to the MBM control, but it might also play some additional paracrine and autocrine functions which are very likely the basis for explaining the great number of pathophysiological effects related to conditions different from the MBM-related vitamin D-dependent effects [9,10]. In fact, a great number of studies have suggested that low levels of vitamin D might increase the risk of cancer, diabetes mellitus, infections, autoimmune and immune related disorders, cardiovascular (CV) diseases and even the mortality rate for any cause [11,12,13,14,15,16].

Though calcitriol is a much more potent vitamin D metabolite than calcidiol, the circulating levels of the latter are considered to be the most reliable index of global vitamin D status.

Renal transplantation (KTx), though with good reason considered to be the best therapeutic option for patients with a terminal stage of renal disease, is burdened with many complications related to immune-mediated, CV, neoplastic, infective, musculoskeletal and metabolic problems which still threaten the survival of both the patient and the graft and negatively impact on the quality of life of the KTx recipients. Since vitamin D has been claimed to be potentially involved in all the above listed pathological conditions through its classical and non-MBM related effects, there is no wonder that a great number of papers, focused on the possible impact of vitamin D status on the clinical complications and outcomes in the KTx patients, have been published in recent years.

The main aim of the present review is to critically analyze how much of the alleged effects of vitamin D status in most of the above mentioned fields can be reliably accepted as certain, on the basis of the most recent evidence. We have limited ourselves to dealing with native (nutritional) vitamin D. In particular we have focused on the most recently published evidence which support or contradict the alleged functions of nutritional vitamin D in the KTx clinical setting. For this purpose, in addition to the basic references on these topics, we performed a systematic search (pubmed) of all the papers published in the last ten years which specifically addressed the defined topics, favoring the interventional studies, metanalyses, recent systematic reviews and statistically robust observational studies (as agreed by all three authors of the present review).

Before addressing the issue of vitamin D status in KTx patients, one cannot help but face the limitations in defining the condition of sufficiency of vitamin D both in the general population and, more relevantly for the present paper, in the KTx recipients.

## 2. Limitations in the Assessment of Vitamin D Status

Given that the evaluation of the vitamin D status is based on the assessment of serum levels of 25-OHD, the first limitation is related to the different laboratory methods used in the published studies. In fact, although the most easily available and cheapest methods (automated immunoassays) have experienced a marked improvement, their validation in comparison with the most standardized methodologies (HPLC, liquid chromatography-tandem mass spectrometry, radioimmunoassay) is far from being completely and satisfyingly achieved [17,18,19].

Second, there is great variability in vitamin D levels among different populations or individuals of different ancestral characteristics, due to both genetic and geographical differences [20,21,22,23]. Considering that black Americans have lower levels of vitamin D than white Americans and that different genetic polymorphisms can explain approximately 10% of vitamin D levels [23], the prevalence of vitamin D deficiency could be consistently different in cohorts of subjects from different countries. Furthermore, in the same individuals vitamin D status can change over time due to seasonal variations or to modifications of the lifestyle behaviours (outdoor or indoor activity) [24]. This aspect is particularly relevant in KTx recipients, given the usual prescription of avoiding sun exposure and/or using sunscreens with high protection levels for reducing skin cancer risk.

Third, it still remains to be fully clarified whether the total or the free (unbound to carrier proteins) circulating 25(OH)D should be considered as the reference of the vitamin D status and hence measured. In fact, it is well recognized that vitamin D circulates mainly bound to DBP and to a far lesser extent to albumin, with its free circulating fraction representing only 0.1% of the total circulating amount. Since it is well recognized that vitamin D activity is mainly dependent on its free form, it has been suggested that vitamin D status should be based on the assessment of the free fraction and not of the total circulating vitamin D. This point could be of particular relevance in KTx patients, given the potential reduced synthesis of DBP by the liver, due to the catabolic effects of some immunosuppressive drugs (steroids, calcineurin inhibitors, mycophenolate, mTOR) or to the urinary loss of this protein in the presence of relevant proteinuria. However, there are still many unsolved problems regarding the dosage of DBP and of free fraction of vitamin D. Thus, given the relatively good relationship between the free and the total fraction of vitamin D, we can continue to rely on the measurement of the latter [25,26].

A fourth limitation in the assessment of vitamin D status is related to the uncertainties regarding which parameters are informative of the real sufficiency of vitamin D status, thus enabling us to define the requested threshold levels for maintaining vitamin D effects in the “normal” range. It has been suggested that the vitamin D levels that prevent the PTH increase and/or the change in bone status, as assessed by bone histology and/or bone mineral density (BMD) assessment, and/or the occurrence of fractures, can be defined as sufficient. However, great controversy exists on these criteria which have been recently challenged by some authors, since they could push up the threshold of sufficiency, while a more physiological approach should rely on the vitamin D levels associated with the estimated average requirements (EAR) which avoids the above listed clinical outcome [24,27,28,29]. For all these reasons, there is still disagreement on what should be considered “normal” vitamin D levels, with some authors considering 30 ng/mL as the minimal acceptable concentration, while other authors think that the minimal acceptable threshold of 20 ng/mL could be more appropriate, in agreement with more recent statements from the Institute of Medicine (IOM) [30].

On the other hand, the biochemical and clinical criteria for defining a sufficient vitamin D status in the general population are particularly poorly (if at all) informative in the KTx population. In fact, KTx patients often have high PTH levels, an altered bone status and an abnormally high occurrence of fractures mainly as the consequence of their previous (long) history of renal failure [31].

The definition of the sufficiency level of vitamin D became even more confusing with the increasing awareness that vitamin D can play some as yet undefined role also in other non-MBM related physiological processes. At the present time, it is impossible even only to speculate what might be the levels of vitamin D requested for ensuring the supposed non-MBM related effects.

## 3. Epidemiology of the Vitamin D Status in KTx Recipients

The information on the vitamin D status in the recipients of a renal graft is based on relatively few and incomplete studies. In Table 1 we have summarized the main results of those studies which reported on the prevalence of the native vitamin D status in different cohorts of KTx patients [32,33,34,35,36,37,38,39,40,41,42,43,44,45].

Notwithstanding the quite variable criteria used for the categorization of the deficiency/insufficiency status, it appears quite evident that most KTx patients have moderately or even severely depressed levels of native vitamin D. In particular, some cohorts which included a consistent proportion of Afro-American (AA) patients [35] or were from countries with little sun exposure [36] were characterized by a very limited percentage of patients with sufficient levels of vitamin D, however defined, with only 12% or 3–6% of the overall patients having vitamin D levels above 30, respectively. Furthermore, in one of these studies from England [36], including 244 KTx recipients, where 104 of them had received a KTx less than one year previously while the remaining 140 were long-term KTx recipients, the authors found that moderate (calcifediol between 15 and 5) and severe (calcifediol of <5) vitamin D deficiency was more frequently found in the short term group as opposed to the long-term group. Overall, only 3% of the short term and 6% of the long-term KTx recipients had sufficient levels of vitamin D [36]. It is also worth underlining that some difference among the cohorts could be explained by a different attitude of the transplant centers to use vitamin D supplementation. In fact, in a cross-sectional study from a Danish group, including 173 KTx patients, a slightly lower percentage of patients with vitamin D deficiency (29%) was reported [37]; however, 69% of women and 51% of men in this KTx cohort received vitamin D supplements. In fact, in a more recent study from Canada, performed in 331 KTx patients, the authors found that vitamin D insufficiency/deficiency (calcifediol of <30 ng/mL) was present in 35.2% and 76.5% of KTx patients who received or did not receive vitamin D supplements, respectively [41].

In addition to the conditions discussed above, many other factors can contribute to the variability in the reported prevalence of vitamin D deficiency/insufficiency in the different cohorts of KTx patients [32,41] (see Table 2). Among them, proteinuria deserves more discussion. In fact, urinary protein loss, which occurs with a certain frequency in KTx patients, is almost invariably associated with the loss of DBP in urine. This could be at least in theory followed by a reduction of the circulating levels of the DBP and of its specific ligand, namely vitamin D, increasing the occurrence of a deficiency/insufficiency status [46] However, the reduction of the total circulating amount does not necessarily translate into a real deficiency/insufficiency condition, since the free (active) fraction of vitamin D can still remain within the normal range. Furthermore, even the fall in the total circulating amount of vitamin D has been recently questioned by Doorenbos and coworkers [47], who, though confirming that proteinuria is invariably associated with a consistent urinary loss of the DBP, have not observed any significant change in both DBP and vitamin D total or free circulating levels.

The available epidemiological data show that low levels of vitamin D are frequently found in KTx patients and supplementation with native vitamin D can improve, though only to a limited extent, the vitamin D insufficient/deficient status.

## 4. Vitamin D Status and Mineral and Bone Disease in KTx

It is well known that the derangements of mineral (MM) and bone metabolism are frequent complications in KTx patients [48,49]. In fact, persistent secondary hyperparathyroridism (PSHP) is frequently observed after KTx and it has been claimed to be one of the causal factors for the increased incidence of skeletal fractures in KTx recipients [31,50,51]. Furthermore, PSHP of KTx patients is frequently associated with hypercalcemia, which has been suggested to contribute to both graft dysfunction and progression of vascular calcifications in such patients [52,53].

It is widely recognized that the reduced bioavailability of vitamin D can directly and indirectly stimulate PTH production, negatively affecting the musculoskeletal system [34,54,55].

Given the reported high prevalence of vitamin D insufficiency/deficiency in KTx patients, a priority question is related to whether and how much the deficit of native vitamin D can contribute to the MM and bone diseases and, even more important, if its correction could positively impact on bone health in this clinical setting.

Though the main causal factors of PSHP are related to the degree of secondary hyperparathyroidism (SHP) preceding the KTx, a suboptimal vitamin D status might contribute in determining a higher level of PSHP. Thus, the correction of vitamin D insufficiency/deficiency might contribute to a better control of PSHP after KTx and possibly to an improvement of the related bone disease.

In most of the few interventional studies directed to prevent bone loss and fractures occurring in KTx patients, the proposed therapy consisted of bisphosphonates, active vitamin D metabolites, calcium supplements or cinacalcet [56,57].

Few studies have addressed this topic investigating the effects of native vitamin D supplementation on the MM-related parameters and/or bone health in KTx recipients.

In a study from France, 49 KTx patients, with calcifediol levels lower than 30 ng/mL, were treated with cholecalciferol 100,000 IU every two weeks from the fourth to the sixth month after KTx and thereafter with 100,000 UI every other month, while 47 KTx patients with the same degree of vitamin D insufficiency did not receive any vitamin D supplement. After one year, the calcifediol levels significantly increased with a concomitant decrease of PTH levels in the treated group, while no significant change was observed in the untreated patients [58]. In another study from Spain, the authors randomised 168 KTx to receive 266 mcg of calcifediol by oral route either monthly or biweekly. Both regimens were effective in correcting the vitamin D insufficiency in most patients with a significant reduction of PTH [59].

In a study by Wissing and coworkers, 91 KTx patients were randomized to receive calcium supplementation with or without the addition of a monthly dose of 25,000 IU of cholecalciferol. Although in this study, vitamin D supplementation was also associated to a reduction of PTH levels, no significant effect was observed as far as bone mineral loss [60].

To the best of our knowledge, there is no study which faced the issue of the possible effect of the correction of vitamin D insufficiency on skeletal fracture occurrence. In a recent retrospective observational study, two groups of patients who received a KTx in a single center in two different periods of time, from 2004 to 2006 and from 2009 to 2011, were compared. The authors found that in the group transplanted in the most recent period, vitamin D supplementation increased and this finding was associated with a reduction in the percentage of vitamin D deficiency from 64 to 20%, and a concomitant decrease of fracture incidence from 9.1 to 3.1% [61]. However, given the retrospective and observational design of this study it cannot be considered to be proof that vitamin D supplementation can reduce fractures in KTx.

It is also worth considering that the doses and the schedules used for correcting vitamin D insufficiency/deficiency were quite variable among these quoted studies, which makes it difficult to draw any definite conclusions.

Collectively, these studies of KTx patients suggest that the correction of native vitamin D levels could contribute to a better control of PSHP; however, no effect on bone health has yet been demonstrated.

## 5. Vitamin D Status and Potential Non-MBM Related Effects in KTx

As mentioned before, it has been suggested that vitamin D plays a role in a number of non-classical biological pathways due to the almost ubiquitary expression of its specific receptor and to the capacity of a great number of tissues utilizing 25(OH)D to synthesize calcitriol. In recent years, there has been a flourishing of experimental and clinical papers suggesting a number of potentially beneficial effects of vitamin D in a vast array of different pathological conditions. Most of these postulated effects may play a counteracting role towards both the most common complications occurring in KTx patients and most of the factors which concur to allograft dysfunction. It is out of the focus of the present review to address the long list of studies (mostly experimental and observational) which dealt with the alleged non-classical “pleiotropic” effects of vitamin D (this topic has been recently reviewed [62]).

In the following paragraphs, we will limit ourselves to summarizing the results of the studies recently published on this topic, dealing separately with the potential effects on the general clinical complications and those potentially affecting the graft outcome.

### 5.1. Potential Effects Counteracting KTx Complications

**Figure 1:**
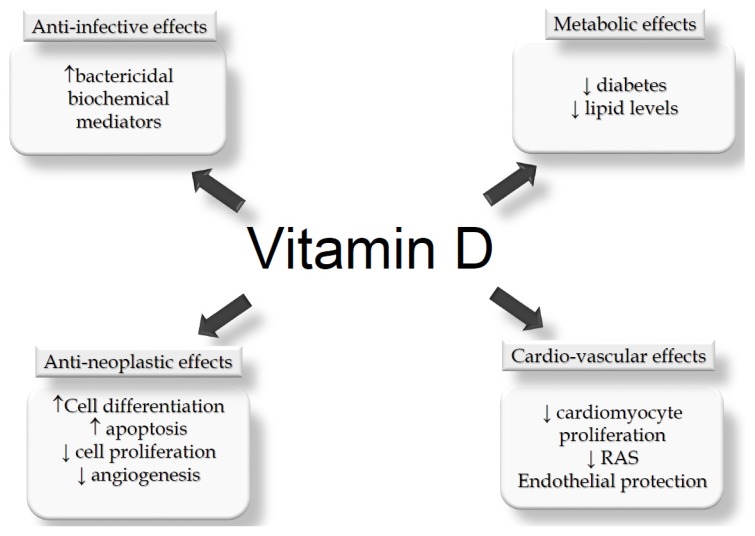
Suggested “non-mineral and bone metabolism (MBM) related” effects of native vitamin D which could play a beneficial role on the main clinical complications which occur in kidney transplant (KTx) patients. RAS = renin angiotensin system.

Even though KTx recipients generally achieve far better results than dialysis patients, they are nevertheless subject to numerous complications which negatively impact on both survival and quality of life. Among the long list of complications which can occur in KTx patients, cardiovascular, infectious, neoplastic and metabolic diseases are the most relevant ones. In fact, a vast number of studies suggest that vitamin D deficiency may be involved in or add to the occurrence of these clinical events (Figure 1) [62].

After the seminal study from Li and coworkers [63], who showed that vitamin D can play an inhibitory effect on the Renin-Angiotensin system, a number of experimental and observational studies further supported a potentially beneficial role of vitamin D in preventing cardiovascular complications in many clinical settings of chronic kidney diseases, including KTx (we recently revised this topic; [15]).

Another potentially beneficial effect of vitamin D in the KTx clinical setting is related to its hypothesized protective effects against infections. As an indirect support of this hypothesis, in a retrospective evaluation of 89 KTx recipients, the authors found that vitamin D insufficiency (<20 ng/mL) was associated with an increased incidence of opportunistic infections [64].

The potential impact of vitamin D levels on one of the most frequent metabolic complications of KTx patients was recently supported by a prospective observational study carried out in a single KTx transplant center, demonstrating that vitamin D deficiency (defined as 25(OH)D <10 ng/mL) was found to be associated with an increased risk of occurrence of post-transplant diabetes mellitus (PTDM) [44].

A possible link between low pre-transplant levels of native vitamin D and the risk of developing cancer after KTx has been suggested by an observational study performed in 363 KTx patients [65]. In fact, the authors reported that the risk of cancer increased by about 12% for each ng/dl decrease of vitamin D levels, which was double the increase of the risk associated with increasing age of the patients (6% for each additional year).

On the other hand, recent evidence, based on randomized clinical trials and metanalysis, has mitigated the enthusiasm about most of these alleged “pleiotropic” effects of vitamin D, since no clear beneficial effects of vitamin D supplementation has been demonstrated on the prevention of neoplasia, CV and/or metabolic complications in many clinical settings [39,66,67,68,69,70,71].

Thus, although many experimental and epidemiological data suggest a potentially beneficial role of vitamin D in many pathological conditions, there is still no evidence that the correction of vitamin D insufficiency/deficiency can improve any clinical outcome.

However, it should be stressed again that most of this evidence has been produced in clinical conditions different from KTx, which still continues to lack consistent studies and hence of evidence.

### 5.2. Potential Effects Counteracting Allograft Dysfunction

**Figure 2:**
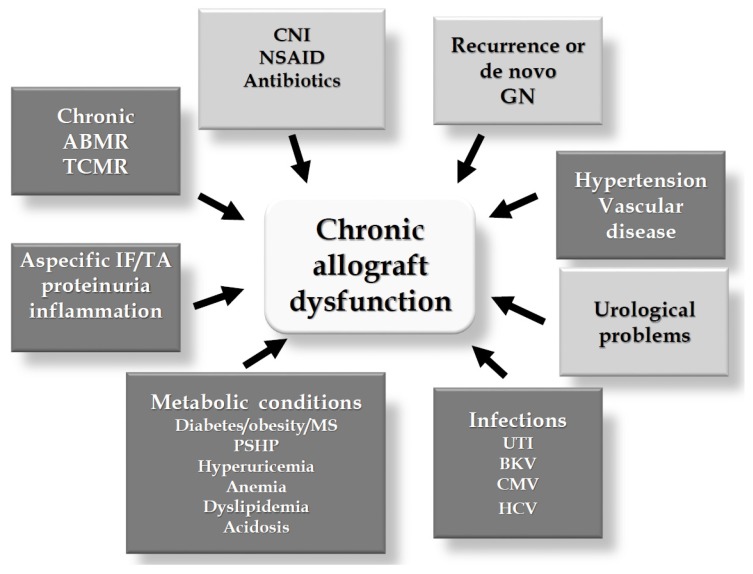
Main factors contributing to the development of chronic renal allograft dysfunction. The darker boxes represent mechanisms on which vitamin D status could play some counteracting role. ABMR = antibody mediated rejection; BKV = BK virus; CMV = cyomegalovirus; CNI = calcineurin inhibitors; GN = glomerulonephritis; IF/TA = interstitial fibrosis/ tubular atrophy; HCV = hepatitis C virus; MS = metabolic syndrome; NSAID = non steroid anti-inflammatory drugs; PSHP = persistent secondary hyperparathyroidism; TCMR = T-cell mediated rejection; UTI = urinary tract infections.

One of the main and yet unsolved problems in the KTx field is related to chronic allograft dysfunction(s) (CAD) which still represent the major limitation to graft survival in the long-term. A large number of factors may contribute to CAD, the most relevant of which are shown in the Figure 2. Vitamin D deficiency has been shown to be associated with most of these factors, potentially contributing to the occurrence of CAD (darker boxes in Figure 2). Most of these potentially beneficial effects have been dealt with in recent reviews [62,72].

Particular attention has been paid in recent years to the potential immunomodulatory effects of vitamin D, since most of the cells involved in the innate (monocytes, dendritic cells) and in the adaptive (T-cell, B-cell) express both CYP27B1 and VDR, indicating their capability of both synthesizing calcitriol from 25(OH)D and of responding to its effects by autocrine/paracrine pathways, with their activity being modulated by vitamin D. Collectively, the final effect of vitamin D on the immune system could be the shift toward a less inflammatory and a more tolerogenic phenotype, playing a potentially positive role on graft survival [72,73,74,75,76].

In a former study performed in 64 KTx patients who were submitted to protocol biopsy, Courbebassie et al. reported no association between cholecalciferol supplementation and the histologic (Banff scores) and functional (iohexol clearance) indices of graft function [77]

However, Bienaimé et al. subsequently studied a larger cohort of 634 KTx patients, reporting that basal low levels of 25(OH)D were predictive of a lower glomerular filtration rate (GFR) at one year after KTx and were associated to a greater degree of interstitial fibrosis and tubular atrophy in protocol biopsies performed 3 and 12 months after transplantation [40].

A former study carried out in 106 KTx patients in whom a pre-transplant measurement of 25(OH)D was available, showed that vitamin D levels were significantly related to the GFR levels during the first three years after KTx [78]. In another more recent study carried out in 435 KTx patients followed up over a mean of seven years, Keyzer and colleagues reported that very low levels of native vitamin D (<12 ng/mL) were associated with increased mortality and a more pronounced reduction of GFR [45]. In the same direction, a prospective observational study performed in 264 Japanese KTx patients showed that vitamin D insufficiency/deficiency was associated with a higher decline of GFR during the first 10 years after KTx, but not thereafter [43].

The risk of developing an acute rejection (AR) in relation to vitamin D status was evaluated in a US cohort of 351 KTx recipients. In this study, the authors reported that patients with vitamin D levels of <20 ng/mL, assessed within 30 days after KTx, had a more than double risk of AR than patients with normal vitamin D levels [42].

In an observational study on 516 KTx patients, low 25(OH)D levels were associated with worse renal outcomes, and supplementation with cholecalciferol were associated with both better renal and patient outcomes [79].

Collectively, all these data seem to suggest that it could be worth correcting vitamin D insufficiency/deficiency in KTx patients. However, it should be underlined that all these studies are observational and often retrospective studies, so their informative level is very limited. We need to wait for the results of the few ongoing randomized controlled trials so as to be able to draw more definitive conclusions on this issue [80,81,82]. However, some of these trials started a long time ago and/or are not yet recruiting patients, so it is highly unlikely we will obtain more evidence in the near future.

## 6. Conclusive Remarks

In the last few years, the renal transplant community has become more aware that native vitamin D insufficiency/deficiency is very common in KTx recipients. Its correction can be at least in part achieved by native vitamin D supplementation.

Though we are well aware that such a correction can contribute to a better control of the levels of PTH, which are often elevated in these patients, the beneficial effects of this therapeutic intervention on bone health remain to be defined.

Even more undefined are the possible beneficial effects, if any, of the correction of vitamin D deficits on the general medical complications often occurring in KTx recipients, as well as on the alleged protective effect on renal grafts.

It is also worth underlining that there is still no general agreement on the optimal target of vitamin D to be achieved, the doses which should be used, and how long the supplementation should be maintained.

On the other hand, when using any vitamin D metabolite in KTx recipients we should also be aware of the risk of inducing hypercalcemia which could be more frequent than in the general population. In fact, KTx patients are more prone to developing hypercalcemia, due to the synergistic effects of the PTH levels being disproportionately high compared to the renal function, associated with the often low phosphorus levels and the increased sensitivity of bone and intestine to PTH and vitamin D, respectively [52]. Hence, overzealous vitamin D repletion might be associated with an increased risk of hypercalcemia in KTx patients with the potential risk of negatively affecting the graft function [83].

The risk of hypercalcemia could be further increased in the case of (unrecognized) polymorphic mutations of the enzymes involved in the metabolism of vitamin D metabolites [84,85].

We still need to wait for further definitive evidence, but in the meantime we could “moderately” correct vitamin D levels in KTx recipients while carefully monitoring serum calcium levels.

## Figures and Tables

**Table 1:** Original studies reporting data relative to the native vitamin D status in KTx patients.

References	N/tot	Gender M %	Ethnicity %	Age Years m ± sd or (R) or (IQR)	KTx Vintage Years m ± sd or (R) or (IQR)	Country	Vitamin D Status % of Patients According to 25(OH)D Levels (ng/mL)
[33]	31/n.r.	54.8	n.s.	(R 10–75)	7 (R 0.5–19)	Germany	48.3% < 15
							48.5% 15–30
							3.2% > 30
[34]	419/n.r.	62.8	n.s.	51.0 ± 15	7.2 ± 6.4	Canada	27.2% < 16
							48.2% 16–30
							24.5% > 30
[35]	112/134	64.0	Cauc 64.3	51.6 ± 13.1	Assessed at time of KTx	United States	28.6% < 10
			AA 24.1				58.9% 10–29
			Other 11.6				24.5% > 30
[36]	244/320	61.9	Cauc 95	46.1 (R 21–76)	Short term (N. 104) 0.28 (R 0.16–0.98)	United Kingdom	68% < 16
							29% 16–30
			Asian 3.7				3% > 30
					Long term (N. 140) 6.0 (R 1–24)		51% < 16
			Black 1.3				43% 16–30
							6% > 30
[37]	173/242	49.9	Cauc 91	53.4 ± 11.7	7.4 (IQR 3.3–12.7)	Denmark	51% < 16
							29% 16–30
			Black 9				
							20% > 30
[38]	111/n.r.	58.8	n.s.	50.5 ± 11.5	6.7 ± 5.1	Italy	69.1% ≤ 30
							21.9% > 30
[39]	331/389	61.6	n.s.	52.2 ± 14.1	n.r.	Spain	28.7% < 16
							48.6% 16–29
							22.7% > 29
[40]	634/n.r.	58.7	n.s.	48.3 ± 13.4	n.r.	France	54.9% < 15
							36.8% 15-30
							8.3% > 30
[41]	331/717	51.1	Cauc 85.8	51 (IQR 41.5–60.2)	6.7 (IQR 2.9–10.8)	Canada	45.3% ≤ 30
			Other 14.2				54.7% > 30
[42]	351/1211	63	AA 22	52.3 ± 13.6	n.r.	United States	61.5% ≤ 20
			Other 78				38.5% > 20
[43]	264/n.r.	61.3	n.s.	49.0 ± 12.3	10.4 (R 2–18)	Japan	24.2% < 12
							44.7% 12–20
							31.1% > 20
[44]	444/1083	60.6	Cauc 89.2	50.9 ± 13.7	4.0 (R 0–11)	France	19.8% < 10
							59.5% 10–30
			Other 10.8				
							20.7% > 30
[45]	435/847	51	n.s.	52 ± 12	6.3 (IQR 3.1–11.7)	The Netherlands	49% < 20
							33% 20–30
							18% > 30

Footnotes: N/tot = KTx patients included in the study/overall KTx cohort; M = male patients; n.r. = not reported; n.s. = not specified; m ± sd = mean ± standard deviation; R = range; IQR = interquartile range; Cauc = Caucasian; AA = Afro-American.

**Table 2:** Factors which can play a role in the different prevalence of the vitamin D deficiency/insufficiency status in KTx patients (neg = increases the risk for the finding of low total vitamin D levels; pos = reduces the risk for the finding of low vitamin D levels; BMI = body mass index; KTx = kidney transplantation) [32,41,46,47].

Factor	Characteristics	Type of Effect
Ethnicity	Afro-Americans	neg
Age	Elderly	neg
Gender	Women	neg
BMI	High	neg
Smoking	Yes	neg
Sun exposure	Yes	pos
Dietary intake/VitD supplements	Yes	pos
Diabetes	Yes	neg
Liver dysfunction	Yes	neg
Urinary protein	High	neg (?)
Time from KTx	Early	neg
Steroid doses	High	neg
